# A Prospective Observational Cohort Study for Newly Diagnosed Osteosarcoma Patients in the UK: ICONIC Study Initial Results

**DOI:** 10.3390/cancers16132351

**Published:** 2024-06-27

**Authors:** Alexa Childs, Craig Gerrand, Bernadette Brennan, Robin Young, Kenneth S. Rankin, Michael Parry, Jonathan Stevenson, Adrienne M. Flanagan, Rachel M. Taylor, Lorna Fern, Dominique Heymann, Filipa Vance, Jenny Sherriff, Saurabh Singh, Rubina Begum, Sharon L. Forsyth, Krystyna Reczko, Kate Sparksman, William Wilson, Sandra J. Strauss

**Affiliations:** 1 University College London Hospitals NHS Foundation Trust, 250 Euston Road, London NW1 2PG, UK; 2 The Royal National Orthopaedic Hospital Trust, Brockley Hill, Stanmore HA7 4LP, UK; 3 Royal Manchester Children’s Hospital, Oxford Road, Manchester M13 9WL, UK; bernadette.brennan@mft.nhs.uk; 4 Sheffield Teaching Hospitals NHS Foundation Trust, Weston Park Hospital, Whitham Road, Broomhall, Sheffield S10 2JF, UK; 5 Newcastle Centre Cancer, Paul O’Gorman Building, Framlington Place, Newcastle upon Tyne NE2 4AD, UK; 6 The Royal Orthopaedic Hospital NHS Foundation Trust, Bristol Road South, Northfield, Birmingham B31 2AP, UK; 7 UCL Cancer Institute, London WC1E 6DD, UK; 8 Sarcoma Research Unit, Department of Oncology & Metabolism, University of Sheffield, Beech Hill Road, Sheffield S10 2RX, UK; 9 Nantes Université, CNRS, UMR6286, US2B, Institut de Cancérologie de l’Ouest, 44800 Saint-Herblain, France; 10 ICONIC Patient Representative; 11 Queen Elizabeth Hospital Birmingham, Mindelsohn Way, Edgbaston, Birmingham B15 2GW, UK; 12 Centre for Medical Imaging, University College London, London WC1E 6BT, UK; 13 CRUK & UCL Cancer Trials Centre, University College London, 90 Tottenham Court Road, London W1T 4TJ, UK

**Keywords:** osteosarcoma, observational, biomarkers, treatment, quality of life

## Abstract

**Simple Summary:**

Treatment for osteosarcoma (OS) has remained largely unchanged over the past 25 years. This is due in part to the low incidence of the disease, which makes the design of and recruitment to clinical trials challenging. The aim of our ongoing study is to establish a platform for the recruitment of newly diagnosed OS patients across the UK, with corresponding collection of clinical and patient-reported outcome (PRO) data, as well as biospecimen sample collection. In Stage 1 of the study, we established the feasibility of patient recruitment and patient data and sample collection. In Stage 2, biological and clinical data will be correlated with outcomes to develop prognostic biomarkers and inform the development of future clinical trials.

**Abstract:**

There has been little change to the standard treatment for osteosarcoma (OS) over the last 25 years and there is an unmet need to identify new biomarkers and novel therapeutic approaches if outcomes are to improve. Furthermore, there is limited evidence on the impact of OS treatment on patient-reported outcomes (PROs). ICONIC (Improving Outcomes through Collaboration in Osteosarcoma; NCT04132895) is a prospective observational cohort study recruiting newly diagnosed OS patients across the United Kingdom (UK) with matched longitudinal collection of clinical, biological, and PRO data. During Stage 1, which assessed the feasibility of recruitment and data collection, 102 patients were recruited at 22 sites with representation from patient groups frequently excluded in OS studies, including patients over 50 years and those with less common primary sites. The feasibility of collecting clinical and biological samples, in addition to PRO data, has been established and there is ongoing analysis of these data as part of Stage 2. ICONIC will provide a unique, prospective cohort of newly diagnosed OS patients representative of the UK patient population, with fully annotated clinical outcomes linked to molecularly characterised biospecimens, allowing for comprehensive analyses to better understand biology and develop new biomarkers and novel therapeutic approaches.

## 1. Introduction

OS is a rare cancer, but one of the most common bone sarcomas, with an annual incidence of 0.3/100,000 [1]. Age-specific incidence rates exhibit a bimodal distribution pattern, with the first and highest peak in adolescence (0.8–1.1/100,000 per year) and a significant second peak in patients over 60 years of age. Patient outcomes have remained relatively static over the last 25 years, with 5-year survival rates around 55% across all ages [2]. Adverse prognostic factors include metastatic disease at presentation, axial or proximal extremity tumour site, large tumour volume, older age [3], and blood-borne markers such as elevated serum alkaline phosphatase (ALP), lactate dehydrogenase (LDH), and C-reactive protein (CRP) [3], with 5-year relative survival rates significantly higher for patients aged < 40 years compared to older patients > 40 years (25%) [4,5,6].

There is no single standard chemotherapy regimen for OS internationally. However, there is consensus on multimodal treatment, including multi-agent chemotherapy and resection of the primary site and metastasectomy where appropriate [7]. Combining chemotherapy with surgery for high-grade, localised OS increases disease-free survival (DFS) from <20% to >60%, but with significant associated acute and long-term toxicity [8]. Protocols for localised disease incorporate chemotherapy prior to and following definitive surgical intervention, allowing the assessment of histological response to chemotherapy, which is predictive of overall survival [4], although there are no data to support altering chemotherapy as a result of this [9]. The MAP regimen (doxorubicin–cisplatin–high-dose methotrexate) is the standard of care for patients aged < 40 years in the UK and across many other European countries [9], but delivery of high-dose methotrexate can be challenging in older patients and there is no agreed standard of care across Europe for patients >40 years. Moreover, despite second-line treatment, the prognosis of those with recurrent disease remains poor, with a 5-year post-relapse survival rate of <20% [10,11].

The rarity of OS makes the design of and recruitment to clinical trials challenging. The most recent randomised trial, EURAMOS-1 (NCT00134030), was an international collaborative effort run by four study groups. Despite recruitment of over 2000 patients, there were no changes made to the standard of care in OS. Consequently, there remains an unmet need to identify biomarkers and novel therapeutic approaches to improve outcomes. OS clinical trials to date have focused on a limited number of outcomes, usually in younger patients with localised extremity disease. Thus, addressing complex interrelated questions has not been possible and several subpopulations have been excluded from studies, thereby limiting opportunities to improve the standard of care. These include: those with widely metastatic disease; less common anatomic locations, such as pelvis, spine, and craniofacial bones; those arising against a background of skeletal dysplasia or underlying genetic predisposition; and finally, the significant proportion of OS arising in patients aged over 40 years. Little is currently known about factors influencing treatment decisions in this population or the consistency with which a standard of care is applied. The effect of treatment on quality of life, patient-reported outcomes, and other performance indicators is not well described or understood either. Overall, there is a need to broaden the ambition and scope of OS research while improving access for all patients.

Following the EURAMOS-1 trial, a recommendation was made for national or cooperative study groups to work internally on hypothesis-generating studies with a view to future wider global evaluation. Concurrently, the limited progress in OS outcomes was also identified by the UK National Cancer Research Institute Sarcoma Clinical Studies Group, leading to it being prioritised as an area of unmet clinical need. This has led to the development of the ICONIC study (Improving Outcomes through Collaboration in Osteosarcoma; NCT04132895), a prospective observational cohort study aiming to recruit patients with newly diagnosed OS across the UK, with annotation of clinical data linked to molecularly characterised biospecimens. This platform allows longitudinal collection of clinical and patient-reported data and tissue and blood samples with a view to addressing clinical and biological questions and making continuous analysis and data interrogation feasible to generate hypothesis-driven questions promptly for further evaluation. This article reports preliminary results from the first 102 patients recruited.

## 2. Materials and Methods

### 2.1. Design and Objectives

ICONIC is a prospective observational cohort study that aims to recruit a cohort of newly diagnosed OS patients of all ages across the UK with longitudinal clinical annotation and matched biological samples. Treatment is given as per standard of care, and no treatments or clinical assessments are specified by the study protocol. A key objective is to ensure ICONIC is a collaborative endeavour involving sarcoma sites across the UK to allow inclusion of all eligible patients.

The study was designed to be conducted in two stages. In Stage 1, the aim was to establish the feasibility of patient recruitment and biospecimen collection, with initial biomarker validation in a subset of patients, as well as mechanisms for rapid interrogation of the clinical data. A primary objective was to open at least 15 sites across the UK with representation across all age groups, with a target recruitment of five patients per month.

### 2.2. Participants

Patients are eligible for inclusion if they fulfil these criteria: a new histological diagnosis of OS, or in the absence of osteoid seen on biopsy, pathology and imaging supportive of a diagnosis of OS and registration within 4 months of initial diagnosis. All patients must provide written informed consent, or where applicable, written confirmation of consent from a parent, legal guardian, or person with duty of care.

### 2.3. Data Collection

#### 2.3.1. Clinical Data

All eligible patients have clinical data collected from standard-of-care assessments at baseline within 28 days of registration, including patient characteristics, comprising age at time of registration, gender, height and weight, WHO performance status (patients ≥ 16 years old) or Lansky performance status (patients < 16 years old), and results of baseline bloods (haemoglobin, absolute neutrophil count (ANC), lymphocytes, platelets, creatinine, CRP, albumin, ALP and LDH). A full medical history is documented to include any history of cancer, relevant medical history, presenting signs and symptoms and their duration, causative risk factors (family history, previous radiotherapy, Paget’s bone disease), and smoking status. Data are also collected on tumour characteristics on diagnostic imaging comprising: imaging modality, site, size and location of primary tumour, as well as other relevant findings, including pathological fracture, involvement of neurovascular bundle and intra-articular location. Staging imaging modality used to detect the presence, location, and size of metastases is also collected. Pathology details from diagnostic tissue including tumour grade and histological subtype are also recorded.

Further clinical data are collected at relevant time points during treatment, including at the end of neoadjuvant chemotherapy/prior to surgery, on completion of adjuvant chemotherapy, after radiotherapy, and on completion of all treatment. Details of all administered chemotherapy and radiotherapy are recorded, with note made of initial drug doses (and fractionation where relevant) as well as documented reasons for any dose reductions, delays or discontinuation of therapy, and the addition of adjunctive therapies (e.g., mifamurtide). Full details of all surgical procedures are also collected, including type of surgery performed and presence of postoperative complications. Complete pathology results from surgical resections are recorded.

Follow-up visits take place according to standard-of-care guidelines for all patients, including those who did not receive or complete treatment. Disease status, survival status, and presence of post-operative complications/late treatment toxicity are recorded.

#### 2.3.2. Research Samples

**Figure 1:**
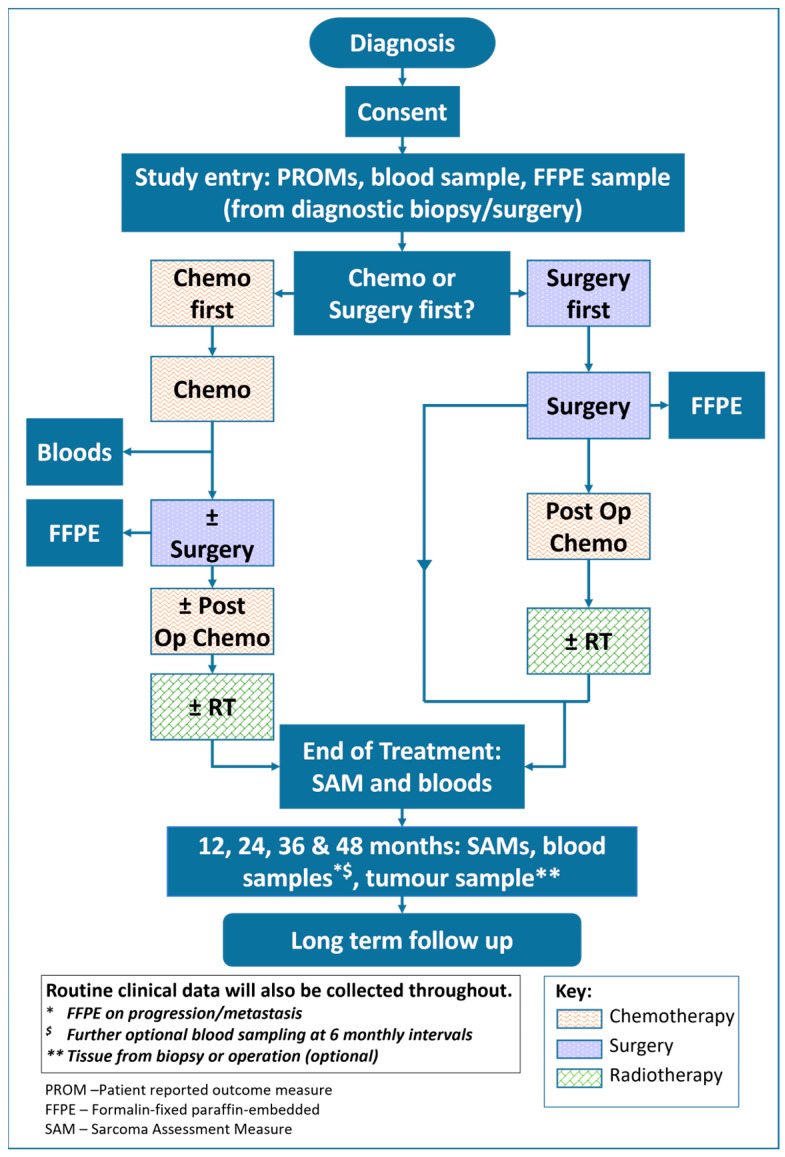
ICONIC trial design.

All patients have whole-blood samples taken for germ-line DNA at baseline, as well as additional optional samples for ctDNA, DNA methylation profiling, and/or future research (Figure 1). Samples are repeated at relevant time points, i.e., during chemotherapy, at completion of treatment, and during follow-up visits (no more than 6-monthly). Where applicable, patients will also have samples repeated at time of disease relapse. For those patients receiving neoadjuvant chemotherapy, optional whole-blood samples are also taken for circulating tumour cell (CTC) analysis at identical time points (baseline and end of neoadjuvant treatment/time of surgery). These will be used to evaluate two CTC isolation and characterisation workflows in OS, namely, Parsortix™ and DEPArray™ versus flow cytometry.

Tissue from diagnostic specimens as well as surgical resections (formalin-fixed paraffin-embedded (FFPE) and fresh frozen according to local policy) will be sent to the Royal National Orthopaedic Hospital (RNOH), Stanmore. Optional study-specific biopsies may be requested at relapse in cases where a biopsy is not routinely collected. For patients in England, frozen tumour tissue will undergo whole-genome sequencing (WGS) when feasible through the NHS England Genome Medicine Service and analysed alongside OS genomes submitted to the Genomics England 100,000 Genomes Project and other existing published datasets [12,13,14,15,16,17,18]. Further analyses may be undertaken, including RNA and epigenetic analyses, as part of the Osteosarcoma Research Consortium funded by the Tom Prince Cancer Trust. FFPE tissue will be used to determine the feasibility of DNA/RNA extraction for validation studies and to validate findings from WGS and other analyses, including molecular targeted sequencing and copy-number analysis.

#### 2.3.3. Patient-Reported Outcomes (PROs)

A sarcoma-specific PRO, the Sarcoma Assessment Measure (SAM) has been developed for use in ICONIC with support from Sarcoma UK and the Bone Cancer Research Trust (BCRT) [19,20,21,22]. It covers the core domains of physical, emotional, social, and financial well-being and sexuality in order to document a comprehensive reflection of quality of life. Patients ≥ 13 years of age are requested to complete the SAM questionnaire along with Toronto Extremity Salvage Score (TESS), EORTC-QLQ-C30 and global rating of change (GRC) at registration, annually, and at relapse. Longitudinal assessment is performed to identify predictors of variance in PROs.

#### 2.3.4. Routes to Diagnosis

To identify the routes and timescales of diagnostic pathways for patients with OS, patients ≥ 13 years are requested to complete a questionnaire at registration detailing symptoms and routes to diagnosis. For patients aged < 13 years, the parent/legal guardian is requested to complete the questionnaire. Questionnaire data from patients and general practitioners (GPs) together with clinical data will be combined to provide a comprehensive description of routes to diagnosis and key time intervals for patients with OS and allow comparisons with other common cancer types [23].

#### 2.3.5. Statistical Analyses

The total expected sample size is assumed to be a minimum of 300 patients based on an average recruitment rate of 5 patients/month across 5 years. Analyses will describe treatment patterns and clinical outcomes of patients treated for OS in the UK and also assess potential predictors of patient outcome. Analyses will use all available data, regardless of final sample size.

#### 2.3.6. Future Analyses

The primary aim of Stage 2 is to investigate and correlate the biological and clinical data with response to chemotherapy and survival outcomes to develop predictive and prognostic biomarkers that can identify patients for specific therapies. Prognostic factors of chemotherapy response will be assessed using logistic regression, and prognostic factors of survival outcomes will be assessed using Cox regression. Both univariate and multivariate analyses will be employed. PRO data will be analysed both descriptively and using mixed modelling approaches to describe longitudinal changes.

## 3. Results

The ICONIC trial opened in October 2019 and Stage 1 completed in July 2021. The preliminary results of patients recruited during Stage 1 are reported below.

### 3.1. Recruitment

Between October 2019 and July 2021, 102 patients were recruited to the ICONIC study from 22 sites across the UK. One patient withdrew from the study soon after being recruited, and the diagnosis was changed from OS to sclerosing epithelioid fibro-sarcoma in one case. Both are excluded from analyses.

The primary Stage 1 objective of recruiting five patients per month once 15 sites were open was achieved with representation from paediatric, teenage and young adult (TYA), and adult oncology sites across the UK. All five surgical centres in England were opened and had recruited patients to the study. Approximately 10% of patients approached for the study have not been entered. The reasons cited for this included patient choice and clinical decision.

### 3.2. Patient Characteristics

Patient characteristics are shown in Table 1. Recruitment appears to reflect the expected demographics for OS, with a median age of 17 years but a wide range of 5–82 years. The study has successfully recruited from older patient populations, typically excluded from prior OS studies, with 9 of 100 (9%) of all patients being over 50 years of age. The majority of patients, 95 of 100 (95%), had symptoms at time of diagnosis, and these were predominantly due to local disease effects, including pain (87/100, 87%), swelling (58/100, 58%), and reduced movement (43/100, 43%), rather than systemic symptoms (weight loss (13/100, 13%), night sweats (5/100, 5%)). Extremity, i.e., femur, tibia/fibula, and humerus, tumours were the most common sites of primary disease affecting 49/100 (49%), 21/100 (21%), and 12/100 (12%) of patients, respectively (Table 1). Importantly, the study has also recruited patients with primary disease presenting at less usual anatomical sites, including craniofacial disease (5/100, 6%), pelvis/sacrum (4/100, 4%), and rib/chest wall disease (4/100, 4%).

### 3.3. Clinical Data

#### 3.3.1. Imaging

MRI was documented as the primary diagnostic imaging modality in 83/100 patients (83%). The majority of patients (99/100, 99%) underwent further staging with CT chest, which excluded thoracic metastases in 64/98 (65%) and demonstrated definitive metastases or indeterminate nodules in 25/99 (25%) and 10/99 (10%), respectively. Preliminary data indicate that methods of skeletal imaging vary across the UK, with whole-body MRI the most frequent modality (49/100, 49%), followed by nuclear medicine bone scan (26/100, 26%) and FDG PET/CT (19/100, 19%), although some patients may have had more than one imaging modality.

#### 3.3.2. Treatment Plan

Data on the intended treatment plan are summarised in Table 2. Multimodality therapy with a combination of surgery and chemotherapy was most common (78/100, 78%), with a small proportion planned for radiotherapy in addition to chemotherapy and surgery (4/100, 4%) and a small number planned for radiotherapy and chemotherapy alone (2/100, 2%). A minority of patients with advanced disease ± poor performance status were planned to receive palliative chemotherapy alone (8/100, 8%). Most patients receiving chemotherapy with curative intent were planned for neoadjuvant treatment (76/100, 76%), with a smaller proportion receiving adjuvant chemotherapy post-surgery alone (16/100, 16%). The intended chemotherapy regimens reflected current standard of care, with the MAP regimen the most common (69/76, 91%) of all neoadjuvant patients and 67/85 (79%) of adjuvant. Mifamurtide was also part of intended adjuvant management in 20/85 (24%) of patients.

#### 3.3.3. Surgery Data

Of 90/100 (90%) patients planned for surgery, limb salvage or radical surgery to axial and craniofacial sites of disease was most common, at 76/90 (84%), with 13/90 (14%) of patients thought likely to require limb amputation according to their surgical centres. Data from patients who have undergone surgery are shown in Table 3. A total of 38/66 (58%) of patients demonstrated a poor response to chemotherapy, defined by post-chemotherapy necrosis <90%. Data regarding the size of tumour, narrowest bone margin, and narrowest resection margin is available for 86/87 (99%), 83/87 (95%), and 75/87 (86%) of patients, respectively.

### 3.4. Research Samples

FFPE tissue is available for all recruited patients and fresh frozen specimens from surgical resection collected in 67/87 (77%) of patients, although the quality of specimens is currently untested. The proportion of frozen samples available from diagnostic biopsies is lower: 24/99 (24%) of patients with primary tumour biopsy. In sum, 23 patients to date have relapsed with metastatic disease, 9 of which have been biopsied.

Overall, 73/98 (74%) of eligible consenting patients have had samples collected for germ-line DNA analysis. For ctDNA and methylation profile studies, 75/98 (77%) have had at least one sample collected, with longitudinal samples for 47/98 (48%, including pre-treatment and at least one other time point). In addition, six patients have had paired samples taken for a pilot study of CTC analysis.

### 3.5. PROs and Routes to Diagnosis

Overall, there has been good uptake for both the patient-reported outcomes and routes to diagnosis questionnaires, with 55/100 (55%) and 71/100 (71%) of the relevant baseline questionnaires completed, respectively. Importantly, we have demonstrated the feasibility of receiving this feedback from GPs, with 73/100 (73%) having returned the routes to diagnosis questionnaire to date.

## 4. Discussion

The ICONIC study was funded in 2018 to provide the infrastructure required to collect clinical data, patient-reported data, and biological samples across all UK OS patients with a view to addressing unanswered clinical and biological questions and developing hypotheses for future interventional trials. The Stage 1 feasibility study was completed in July 2021. The initial recruitment objectives were achieved, with 24 centres open and recruitment of over five patients per month, with patients recruited from all 5 bone tumour surgical centres in England, as well as adult, TYA, and paediatric centres across the UK, including Scotland and Wales. Patient characteristics are in line with previous studies and with representation across all age groups. Despite this, the proportion of patients recruited over 50 years of age is lower than predicted from reported incidence rates (9%), and this will be reviewed in Stage 2 as more patients are recruited. Encouragingly, the proportion of patients approached for the study that have declined entry is low, providing evidence that the study is supported by clinicians and welcomed by patients. In contrast to other OS studies, where fewer common primary sites are often excluded, ICONIC has also recruited patients with more unusual primary sites, including craniofacial disease. This is important if outcomes in surgical and medical management are to be improved for these significant subpopulations. The COVID-19 pandemic impacted cancer services nationally and led to the closure of the central laboratory (Royal National Orthopaedic Hospital, Stanmore) responsible for processing of research blood samples; however, as patients can be recruited within four months of diagnosis, the impact on recruitment for ICONIC was not as significant as that observed for interventional studies, and the study has maintained recruitment in all months since opening. Furthermore, adaptations including a remote consent procedure were rapidly implemented to minimise hospital attendances for patients and enable ongoing recruitment. Despite closure of the central laboratory, the overall number of samples received remained high (76% for germ-line DNA analysis and 60% for additional future research). Genetic predisposition syndromes such as Li-Fraumeni and hereditary retinoblastoma are well-known risk factors for development of OS, with additional potentially pathogenic cancer-susceptibility gene variants being identified using whole-genome or next-generation sequencing [24]. Currently, screening for these syndromes is not the standard of care and their impact on response to chemotherapy and outcome is not well characterised. The ICONIC study offers a valuable opportunity to define incidence in the UK population and potentially perform a correlative analysis of outcomes. Finally, Stage 1 has identified differences in imaging modalities utilised for staging of patients and potential variation in use of mifamurtide across the UK, important observations that are worthy of further analysis in Stage 2.

There are very few studies collecting such comprehensive data for this rare cancer. One study with a similar design is the LEOPARD trial, a prospective observational study that focuses on the prognostic capabilities of ctDNA for patients with bone sarcomas. LEOPARD has recently demonstrated that baseline ctDNA detection is associated with inferior event-free survival for patients aged 1–50 with newly diagnosed localised OS, validating results of previous retrospective analyses. Stage 2 of ICONIC will also examine the prognostic capability of ctDNA, though with the added benefit of including data from older patients and those with more advanced disease, offering the opportunity for comparison and collaboration.

Review of resection pathology reports has shown that these are adequate for reporting of tumour size, bone and soft tissue margins, and response to chemotherapy, providing data to understand the impact of these, as well as the timing of surgery on outcome. A priority for Stage 2 will be to centralise FFPE specimens and determine the quality of these samples and feasibility for biological studies. There has also been successful collection of information regarding fresh frozen tissue, particularly from resection specimens, where 80% of cases have tissue available. The lower rates reported for diagnostic samples may reflect the limited volume of tissue available at biopsy, but will be investigated further in Stage 2 in addition to whether there is geographical variation in the ability to access this. There have been delays in establishing WGS as the standard of care in the NHS due to the COVID-19 pandemic, but this will be a focus for Stage 2 of the study.

Improving the diagnostic experience and development of interventions to address the psychological impact of cancer during and following a cancer diagnosis are national research priorities in the UK and the focus of many charitable initiatives, including the BCRT [25]. Patients with sarcoma may have long complex routes to diagnosis and are more likely to have multiple consultations with their GPs or other health care providers prior to referral onto specialist care units compared to other cancers [26,27,28]. Response rates of the patient and GP routes to diagnosis questionnaire are higher than anticipated. Poorer PROs are recorded by patients with sarcoma in comparison to patients with other cancer types [29,30,31], but there is limited evidence to date on the impact of OS on PROs, quality of life, and other psychosocial outcomes [32,33]. More than 60% of ICONIC participants have returned PROs, indicating that completion of these forms is acceptable to patients. A paediatric version of the SAM is in development (SAM-Paed) and should be available in 2024. This will be incorporated into the ICONIC study, enabling data collection across the full age spectrum of OS patients.

Based on the initial findings of Stage 1, the study has progressed to Stage 2. Additional funding has subsequently been secured to extend recruitment (until 2025), enable longer follow-up, and to make use of the data and samples collected to date to address recognised areas of unmet need in OS research. These include work packages to identify novel OS-specific immune-oncology drug targets and correlate PROs with clinical data over time, as well as contributing data to the wider OS community through engagement with the newly formed European OS consortium FOSTER (Fight Osteosarcoma through European Research [34]. Furthermore, there have been several expressions of interest from the OS community to align research studies, access samples/data, and add ancillary studies to ICONIC, demonstrating additional value of the study.

## 5. Conclusions

ICONIC is the first prospective observational cohort study to recruit UK patients of all ages with newly diagnosed osteosarcoma. Stage 1 has established the feasibility of collecting high-quality clinically annotated data and biological samples, in addition to patient-reported outcome data, in this patient population. It has identified differences in staging and systemic management of patients across the country worthy of further exploration. Stage 2 will examine the association of biological and clinical features with outcomes, with the aim of identifying predictive and prognostic markers to inform development of future clinical trials.

## Figures and Tables

**Table 1:** Patient characteristics at baseline.

Registered Patients	N = 100
**Age, median (range)**	17 (5–82)
**Age**	
Under 16	38 (38%)
16–24	34 (34%)
25–50	19 (19%)
Over 50	9 (9%)
**Stage at diagnosis**	
IA	6 (6%)
IB	6 (6%)
IIA	20 (20%)
IIB	36 (36%)
III	5 (5%)
IVA	12 (12%)
IVB	14 (14%)
Unknown	1
**Sex**	
Female	45 (45%)
Male	55 (55%)
**Cancer signs or symptoms**	**95 (95%)**
Limb/other body part changes	44 (44%)
Swelling	58 (58%)
Redness	6 (6%)
Reduced movement	43 (43%)
Night pain only	9 (9%)
Pain	87 (87%)
Night sweats	5 (5%)
Weight loss	13 (13%)
Other	26 (26%)
**Duration of symptoms**	**N = 95**
1–2 weeks	3 (3%)
3–4 weeks	12 (13%)
5–7 weeks	12 (13%)
2–5 months	47 (49%)
6–12 months	16 (17%)
More than 12 months	3 (3%)
Unknown	2
**Smoking status**	
Never	84 (84%)
Current	6 (6%)
Ex-smoker	10 (10%)
**WHO performance status (age 16+)**	**N = 62**
0	12 (21%)
1	26 (46%)
2	16 (28%)
3	1 (2%)
4	2 (4%)
Unknown	5
**Karnofsky performance status (age < 16)**	**N = 38**
30	1 (4%)
50	2 (7%)
60	2 (7%)
70	4 (14%)
80	4 (14%)
90	10 (36%)
100	5 (18%)
Unknown	10
**Primary imaging type**	
CT	17 (17%)
MRI	83 (83%)
**Site of primary tumour**	
Femur	49 (49%)
Humerus	12 (12%)
Maxilla/mandible	4 (4%)
Metatarsal	1 (1%)
Pelvis/sacrum	4 (4%)
Radius/ulna	3 (3%)
Rib/chest wall	4 (4%)
Scapula/clavicle	1 (1%)
Skull	1 (1%)
Tibia/fibula	21 (21%)
**Imaging to assess extent of disease**	
**CT chest**	**99 (99%)**
No metastases	64/99 (65%)
Metastases confirmed	25/99 (25%)
Indeterminate	10/99 (10%)
**PET scan**	**19 (19%)**
No metastases	13/19 (68%)
Metastases confirmed	5/19 (26%)
Indeterminate	1/19 (5%)
**Isotope bone scan**	**26 (26%)**
No metastases	25/26 (96%)
Metastases confirmed	1/26 (4%)
**WB MRI for metastases**	**49 (49%)**
No metastases	42/49 (86%)
Metastases confirmed	5/49 (10%)
Indeterminate	1/49 (2%)
Report unavailable	1/49 (2%)

**Table 2:** Treatment plan.

Registered Patients	N = 100
**Treatment plan**	
Chemo only	8 (8%)
Radiotherapy with chemo	2 (2%)
Radiotherapy with surgery and chemo	4 (4%)
Surgery only	8 (8%)
Surgery with chemo	78 (78%)
**Chemotherapy plan**	
Neoadjuvant only	7 (7%)
Neoadjuvant + adjuvant	69 (69%)
Adjuvant only	16 (16%)
No chemotherapy planned	8 (8%)
**Neoadjuvant chemotherapy planned**	**N = 76 (76%)**
AP (doxorubicin, cisplatin)	5/76 (7%)
MAP (methotrexate, doxorubicin, cisplatin)	69/76 (91%)
Other	2/76 (3%)
**Adjuvant chemotherapy planned**	**N = 85 (85%)**
AP (doxorubicin, cisplatin)	13/85 (15%)
MAP (methotrexate, doxorubicin, cisplatin)	47/85 (55%)
MAP + mifamurtide	20/85 (24%)
Other	5/85 (6%)
**Surgery planned**	**N = 90 (90%)**
Amputation	13/90 (14%)
Limb salvage	66/90 (73%)
Craniofacial resection	4/90 (4%)
Pelvis/sacrum resection	2/90 (2%)
Rib/chest wall resection	4/90 (4%)
Other	1/90 (1%)

**Table 3:** Surgery data.

Patients Planned for Surgery	N = 90
**Patient had resection of primary tumour**	
Yes	87 (97%)
No	3 (3%)
**Type of surgery**	**N = 87**
Amputation	13 (15%)
Limb salvage	64 (74%)
Reconstruction:	
Implant	54/64 (84%)
Autograft reconstruction	9/64 (14%)
Allograft reconstruction	1/64 (2%)
Craniofacial resection	3 (3%)
Pelvis/sacrum resection	1 (1%)
Rib/chest wall	4 (5%)
Other	2 (2%)
**Maximum tumour dimension—length (mm)**	**N = 87**
Median (range)	117 mm (0–465)
≤80 mm	27 (31%)
>80 mm	59 (69%)
Unknown	1
**Maximum tumour dimension—width (mm)**	**N = 87**
Median (range)	52.5 mm (0–200)
<50 mm	36 (44%)
≥50 mm	46 (56%)
Unknown	5
**Narrowest bone margin (mm)**	**N = 87**
Median (range)	37 mm (0–200)
<50 mm	54 (65%)
≥50 mm	29 (35%)
Unknown	4
**Narrowest soft tissue resection margin (mm)**	**N = 87**
<1 mm	20 (24%)
<2 mm	9 (11%)
≥2 mm	44 (53%)
Tumour at surface of specimen	2 (2%)
Unknown	12
**Post-chemotherapy necrosis** **(among those known to receive neoadjuvant chemotherapy)**	**N = 66**
Median (range)	82.5% (10–100)
<90%	38 (58%)
≥90%	28 (42%)

## Data Availability

Data will be available for sharing after the main trial publication is released. All requests for data will be considered by the chief investigator/TMG, and if approved, shared via a data sharing agreement. Decision-making factors will include: data use is in keeping with patient consent; confidentiality is maintained at all times, i.e., no patient-identifiable data such as date of birth will be provided; data are appropriate for intended purpose; and compliance with legal and ethical requirements is maintained. Any requests for data should be sent to the ICONIC trial team at the Cancer Research UK and UCL Cancer Trials Centre (ctc.iconic@ucl.ac.uk).
